# Combinatorial mutagenesis and fermentation optimization biotechnologies synergistically enhance monacolin K content in functional red yeast rice

**DOI:** 10.3389/fmicb.2025.1669985

**Published:** 2025-08-14

**Authors:** Zhengyan Wu, Chan Zhang, Qing Liu, Song Zhang, Qi Yang, Jun Liu, Dong Lu, Xiang Zhou

**Affiliations:** ^1^Hunan Key Laboratory of Forestry Edible Resources Safety and Processing, Hunan Key Laboratory of Grain-Oil Deep Process and Quality Control, National Engineering Research Center of Rice and Byproduct Deep Processing, School of Food Science and Engineering, Central South University of Forestry and Technology, Changsha, China; ^2^School of Food and Health, Beijing Technology and Business University, Beijing, China; ^3^Biophysics Research Laboratory, Institute of Modern Physics, Chinese Academy of Sciences, Lanzhou, China

**Keywords:** *Monascus purpureus*, monacolin K, mutagenesis, solid-state fermentation, RT-qPCR

## Abstract

**Introduction:**

Red yeast rice (RYR) is produced through solid-state fermentation by Monascus genus. Its functional component, Monacolin K (MK), has the same structure as lovastatin and can effectively inhibit HMG-CoA reductase, thereby reducing serum cholesterol.

**Methods:**

A combinatorial mutagenesis strategy integrating atmospheric room-temperature plasma and heavy-ion radiation was employed to generate mutant strains. The optimizations of substrate components and conditions were carried out during solid-state fermentation (SSF). Subsequently, RYR enriched with MK was produced through scale-up experiments. Additionally, integrated biosynthetic pathway with expression dynamics of MK biosynthetic gene cluster to reveal the efficient biosynthesis of MK in a mutant strain.

**Results:**

A mutant *M. purpureus* CSUFT-1, which exhibited a remarkable 1.67-fold increase in MK production during SSF compared to the original strain, was obtained. Through refinement of SSF parameters, specifically, adding optimized corn steep liquor adjuvant solution [60% (v/v) corn steep liquor, pH modulation at 5, 3 g/L of MgSO_4_·7H_2_O, 1.5 g/L of KH_2_PO_4_] with 20% (v/w) of injection volume, the MK yield was significantly amplified to 32.71 mg/g on day 28. Scale-up experiments confirmed the robustness of the optimized process, with MK production stabilizing approximately at 24.66 mg/g. RT-qPCR results showed that seven key genes, including *mokC* (6.8-fold upregulation) and *mokF* (22-fold upregulation), were significantly activated during the early stage of fermentation to drive precursor synthesis, only *mokE* gene exhibited the sustained overexpression in *M. purpureus* CSUFT-1 during the entire SSF.

**Discussion:**

This work successfully demonstrates a synergistic approach combining advanced combinatorial mutagenesis with precise bioprocess optimization to substantially improve MK yield. The overproducing *M. purpureus* CSUFT-1 and the optimized SSF protocol provide both microbial resource and technical protocol for industrial-scale production of RYR with high MK.

## Introduction

Red yeast rice (RYR), a staple in Chinese culinary culture and a cornerstone in the brewing and fermented food industries, has been utilized for millennia (Su et al., 2003). Its cholesterol-inhibiting component, Monacolin K (MK), exhibits remarkable biological activity, making it highly valuable in various sectors such as food, health supplements, and pharmaceuticals (Kim and Ku, 2018). Beyond its hypolipidemic properties, MK has demonstrated broad therapeutic potential, including anticarcinogenic effects against colon cancer, suppression of acute myeloid leukemia, and neuroprotective activities (Xiong et al., 2019). MK is one of the primary bioactive metabolites synthesized by *Monascus* species, exists in two structural forms: acid and lactone-type (Zhang et al., 2018). In addition, through probe technology, nine genes (*mokA* to *mokI*) related to the MK synthesis were identified out from *Monascus*, thus initiating the era of molecular-level research on the synthesis of MK in *Monascus* (Chen et al., 2008). To precisely enhance the MK content in RYR, efficient breeding techniques was employed to isolate a high-yielding MK mutant strain. Consequently, enhancing MK production is pivotal for advancing the development of MK-based functional products and driving innovation in industrial production (Wen et al., 2020).

In the industrial production of RYR, MK yield is significantly influenced by several factors, including strain, fermentation condition, and substrate composition. Selection of *Monascus purpureus* strains exhibiting high-efficiency MK biosynthesis and citrinin-free traits was conducted to establish high-yield, safe microbial resources for pharmaceutical and nutraceutical industries (Liu et al., 2025). Precise control of fermentation parameters such as temperature, pH, and dissolved oxygen is essential to ensure optimal metabolic activity (Srianta et al., 2021). Additionally, the ratio of carbon sources, nitrogen sources, inorganic salts, and growth factors in the medium directly impacts MK biosynthesis, with optimized medium compositions significantly boosting yield.

In the field of industrial microbiology, integrating physicochemical mutagenesis with fermentation process optimization strategies systematically enhances the synthesis efficiency of secondary metabolites in *Monascus*. At the mutagenesis breeding level, a composite mutagenesis system using Atmospheric and Room Temperature Plasma (ARTP) and Heavy Ion Radiation Facility (HIRFL) has been developed (Bai et al., 2022). The ARTP-generated reactive species triggered the bacterial SOS response, and the selected mutant strain artp-aleBC15 demonstrated significantly enhanced extreme environmental tolerance, achieving a survival rate of 22.4% under acidic stress (pH 2.5) and 0.3% bile salt concentration (Liu et al., 2020). HIRFL mutagenesis, with its high-energy and penetrating heavy ions, causes deeper genetic material damage, increasing MK content in the fermentation broth of *Aspergillus terreus* Z15-7 by 4-fold compared to the original strain (Li et al., 2011). Combining ARTP and HIRFL technologies enables the rapid screening of numerous beneficial mutants, accelerating the breeding process and enhancing efficiency and success rates (Yuan et al., 2020; Zhang et al., 2024). Through this combinatorial mutagenesis approach, the engineered strain DES-26 demonstrated a natamycin titer of 1.64 g/L, representing an 86.36% productivity enhancement compared to the parental strain *Streptomyces natalensis* (Sun et al., 2023). This integrated strategy establishes a robust platform for high-throughput mutant selection and metabolic overproduction optimization.

Fermentation process optimization further leverages these mutagenesis advancements by adjusting conditions such as temperature, pH, and nutrient composition to create an optimal growth and metabolic environment, thereby maximizing yield and quality improvements. In terms of fermentation process regulation, temperature gradient optimization in solid-state fermentation (SSF) of RYR has achieved an MK yield of 9.5 mg/g (Yuan et al., 2023). Compared to commercially available functional RYR with an MK content of 8 mg/g, temperature-regulated SSF processes have increased MK yield to 14.53 mg/g (Liu et al., 2022). When using yam as the fermentation substrate, MK yield was 5.37 times higher than with rice substrate (Lee et al., 2006). In SSF of RYR, initial moisture content (50–55%) and bran content (4.5–5.5%) are crucial for nutrient and oxygen transport (Wu et al., 2023). These metabolic variances create substantial opportunities for functional diversification and broad-spectrum applications of *Monascus*-fermented products through tailored bioprocessing strategies.

In this study, a composite mutagenesis breeding technique was employed to obtain a high-production MK mutant strain. A comparative analysis of substrate-specific fermentation characteristics was conducted to develop functional RYR through SSF. Further, the expression levels of MK biosynthetic gene cluster (*mokA-mokI*) in *M. purpureus* during the SSF process were evaluated to reveal the time-dependent transcriptional dynamics underlying MK biosynthesis. This investigation lays the groundwork for the subsequent production of high-quality RYR enriched with natural monacolins.

## Materials and methods

### Microorganisms and chemical materials

The strain *M. purpureus* M1 was preserved in prof. Chan Zhang’ laboratory at Beijing Technology and Business University. The mutant strain CSUFT-1 (China Central for Type Culture Collection (CCTCC), Wuhan, China, No. M 20241399) was developed through HIRFL using a PRECISIONX-Rad225 irradiator (National Heavy Ion Accelerator Laboratory, Lanzhou, China) and ARTP-M mutant system. Analytical-grade chemicals and laboratory media were obtained from Sangon Biotech (Shanghai, China). Corn steep liquor used throughout this study was purchased from Macklin Biochemical Technology Co., Ltd. (Shanghai, China). The following grain components indica rice, soybean powder, wheat bran, oats were procured from a local market in Changsha (Hunan, China), and the aged indica rice was obtained by storing more than 24 months, the milling-induced broken and indica rice was supplied by Yihai Kerry Arawana Holdings Co., Ltd. (Hunan, China).

### Fermentation medium

1. Liquid-state fermentation (LSF) medium: 30 g/L indica rice powder (80 mesh), 20 g/L peptone and 60 g/L glucose at pH 5.0.
2. SSF medium is shown in Table 1.

**Table 1:** The composition and proportion of the substrate in solid state fermentation.

Medium	Fermentation substrate	Ratio (w/w)
C1	Indica rice: Water (pH 4)	50:5
C2	Indica rice: Rolled oat: Water (pH 4)	50:5:5
C3	Broken indica rice: Water (pH 4)	50:5
C4	Broken indica rice: Soybean powder: Wheat bran: Water (pH 4)	50:1:1:10
C5	Broken indica rice: Soybean powder: Wheat bran: Rolled oat: Water (pH 4)	50:1:2:1:1:10
C6	Aged indica rice: Soybean powder: Wheat bran: Water (pH 4)	50:1:2:1:1:10

### Preparation of spore suspension

*M. purpureus* M1was cultured on potato dextrose agar (PDA) medium at 30 °C for 7 days, after which the mycelium was scraped using a sterile spatula. The mycelium was then transferred to a 100 mL conical flask containing 20 mL of sterile water. The spore mixture was homogenized using a vortex mixer at 2,500 rpm for 3 min to achieve uniform dispersion, followed by sterile filtration through an autoclaved cotton-packed syringe (121 °C, 15 min) to eliminate hyphal debris. The spore concentration was quantified via electron microscope with a hemocytometer and approximately adjusted to 10^7^ spores/mL using sterile water.

### Mutagenesis and screening

Mutagenesis was performed using ARTP and HIRFL techniques, with modifications based on our previous research (Liu et al., 2019b; Bai et al., 2022). Initially, 10 μL of spore suspension of *M. purpureus* M1 was spread onto a sterile steel plate and air-dried on a clean bench. The plate was then transferred to the ARTP system for irradiation fixed at 150 s. Following mutagenesis, 1 mL of seed medium (30 g/L starch, 20 g/L peptone and 60 g/L glucose at pH 5.0) was added to the mutant spore suspension, followed by a 24-h recovery period. Next, the tube containing the ARTP-treated spore suspension was placed in a 35 mm irradiation dish and transferred to the HI (Heavy Ion) system equipped with a carbon ion beam (^12^C^6+^, energy 80 MeV/u, dose rate 20 keV/μm). The sample was irradiated at a dose of 200 Gy. Post-irradiation, the sample was cultured on PDA for 5–7 days, during which mutant strains exhibiting significant differences in colony size, color, and morphology compared to the parent strain were selected. The mycelium was inoculated into a 24-well culture plate (5 mL/well) and incubated at 30 °C in the dark for 4 days. The incubation temperature was then adjusted to 25 °C and maintained for an additional 16 days (Ye et al., 2024). Preliminary screening was conducted by measuring the MK concentration of the broth.

### Mutant strain identification

The morphology of the mycelium was observed using an Asivi CM30W-HK810 biological microscope. To molecularly identify the mutant strain, genomic DNA was extracted from the mutagenized mycelium (Yang et al., 2018). The ITS rDNA region was amplified using universal primers ITS1 (forward) and ITS4 (reverse) through polymerase chain reaction (PCR) (the primers were provided by Sangon Biotech for fungal identification). Homology analysis was performed using the BLASTn algorithm (Basic Local Alignment Search Tool) against the NCBI GenBank nucleotide database. Phylogenetic reconstruction was performed in MEGA (Molecular Evolutionary Genetics Analysis), version 11.0.13 under the Maximum Likelihood (ML) framework. Branch support was assessed with 1,000 bootstrap replicates, and the final tree was midpoint-rooted for visualization.

### Fermentation

Four milliliters of the spore suspension was inoculated into 36 mL seed medium at 10% (v/v) inoculum density. The seed culture was incubated in a rotary shaker at 180 rpm and 30 °C for 72 h.

The fermentation broth (45 mL) was prepared in 250 mL Erlenmeyer flasks, sterilized at 121 °C for 15 min, and aseptically inoculated with 5 mL of seed culture after cooling, with triplicate experimental groups established. The cultures were incubated in a constant-temperature orbital shaker at 30 °C with 180 rpm agitation for 3 days, followed by a secondary fermentation phase at 25 °C and 180 rpm for 17 days to complete the LSF process.

Different varieties of substrates (30 g dry weight) were soaked in pure water at 20–28 °C for 2 h. After filtration, transfer the fermentation substrates to wide-mouth glass bottles (31 × 111 × 58 mm) with 125 mL volume. Subsequently, added 10% or 20% (v/w) pure water (pH adjusted to 4.0 with acetic acid), mixed thoroughly, sealed with 8 layers of gauze and 1 layer of kraft paper. Sterilized in an autoclave at 121 °C for 20 min. After cooling to approximately 40 °C, the substrate was inoculated with 15% (v/w) seed culture and mixed thoroughly under sterile conditions. The substrate was incubated in a dark incubator at 30 °C for 3 days, with care taken to prevent clumping or excessive drying. The temperature was then adjusted to 25 °C, and 0.2% (v/v) acetic acid-water solution was added every 12 h to maintain a proper moisture content.

### Detection of citrinin and monacolin K

The quantification analysis for citrinin followed the High Performance Liquid Chromatography (HPLC) method established in our previous study (Chai et al., 2020). Separation was achieved on a C_18_ column (150 mm × 4.6 mm, 5 μm) with mobile phase A (methanol) and mobile phase B (acetonitrile-isopropanol-0.08 mol/L phosphoric acid, 35:10:55, v/v/v) under gradient elution. The flow rate was maintained at 1.0 mL/min with 10 μL injection volume, column temperature was controlled at 30 °C.

MK standards (analytical grade, Macklin) were prepared as follows: the lactone form (40 mg) was dissolved in 75% (v/v) ethanol-water solution to a final concentration of 40 mg/L, and dissolved in 0.2 mol/L NaOH, ultrasonicated at 50 °C for 60 min, left to stand at room temperature for 60 min to generate 40 mg/L of the acid form of MK. The extraction solution was filtered through an organic microporous membrane with a filtration pore size of 0.45 μm into a liquid phase bottle, and the MK content in the filtrate was detected by HPLC (Ye et al., 2024).

#### 24-well fermentation

Five milliliters of 75% (v/v) ethanol-water solution was added to each well of a 24-well plate containing an equal volume of fermentation broth. The mixture was subjected to ultrasonic extraction for 1 h and then left to stand overnight in the dark to ensure thorough extraction. The extract was filtered through a 0.45 μm organic membrane into an HPLC vial for MK concentration analysis.

#### LSF fermentation

Five milliliters of the fermentation broth was taken, mixed with 15 mL of 75% (v/v) ethanol-water solution, and subjected to ultrasonic extraction for 30 min. The mixture was allowed to stand overnight. Approximately 1 mL of the sample was aspirated using a syringe, filtered and analyzed using the same method.

#### SSF fermentation

A 0.5 g aliquot of the sample powder (100 mesh) was precisely weighed into a 50 mL volumetric flask. Thirty mL of 75% (v/v) ethanol was added, and the mixture was thoroughly mixed. Ultrasonic extraction was performed for 50 min. Additional 75% ethanol was added until the volume approached the flask’s calibration mark, followed by a second ultrasonic extraction for 10 min. The solution was cooled to room temperature and brought to volume with 75% ethanol. The mixture was centrifuged at 4,510 × g for 10 min. The supernatant was collected for analysis.

### Real-time quantitative PCR

For real-time quantitative PCR (RT-qPCR) validation, primers listed in Table 2 were designed using the Primer Quest tool (Integrated DNA Technologies, USA), targeting 10 annotated genes involved in MK biosynthesis. Glyceraldehyde-3-phosphate dehydrogenase (*GADPH*) was used as the reference gene. The procedure followed the same method as in our previous study (Liu et al., 2019a).

**Table 2:** Primer pairs used for the amplification of the MK biosynthetic gene cluster.

Genes	Primer pairs (5′ to 3′)	Length (bp)
*mokA-F*	GACCTCGGTCATCTTGGC	18
*mokA-R*	TTGTTCCAAGCGGTCTTC	18
*mokB-F*	AAACATCGTCACCAGTCT	18
*mokB-R*	CTAAGTCGGGCATCTACC	18
*mokC-F*	CAAGCTGCGAAATACACCAAGCCTC	25
*mokC-R*	AGCCGTGTGCCATTCCTTGTTGTCC	25
*mokD-F*	TTCATCTGCTGCTGGTAT	18
*mokD-R*	AACTTCTCACCGTCAATG	18
*mokE-F*	ATCGCAGGTCACGCACATCCAAGTC	25
*mokE-R*	GTAAAGGCAGCCCGAGCAGCTTCAT	25
*mokF-F*	GAGATCATAGTGGCCGACTGAA	22
*mokF-R*	ACCGTCTCATCCAACCTCACGA	22
*mokG-F*	CCAGGTAACCAACGGATTA	19
*mokG-R*	GATCAGAGCAGTCACCAG	18
*mokH-F*	CAGGAAATCTGGACTTACCCCATTG	25
*mokH-R*	TGTTGGATTGTTGTTGGAGATATAC	25
*mokI-F*	ATGTTGAATGGCAATGATGG	20
*mokI-R*	CAGCGTGGGTGATGTATC	18

### Prediction of the subcellular localization of monacolin K synthetic protein

Protein sequences of the biosynthetic gene cluster of MK (MokA-MokI) from *M. purpureus* strain were collected from the NCBI database (https://www.ncbi.nlm.nih.gov/protein/?term=monacolin%20K, accessed on June 14, 2025). The FASTA-formatted sequences of the MK protein family were submitted to various online servers for subcellular localization prediction: WoLF PSORT (https://wolfpsort.hgc.jp/), Cell-PLoc (http://www.csbio.sjtu.edu.cn/bioinf/Cell-PLoc-2/) and Uniprot (www.uniprot.org). The transmembrane structures of these proteins were analyzed through TMHMM Server v. 2.0 (http://www.cbs.dtu.dk/services/TMHMM-2.0/).

### Statistical analysis

All experiments were performed in triplicate. Data are presented as mean ± standard deviation (SD) and analyzed using one-way ANOVA followed by Dunnett’s multiple comparison test. A significance level of α = 0.05 was applied, with *p* < 0.05 considered statistically significant, * *p* < 0.05, ** *p* < 0.01, *** *p* < 0.001 compared to the control. Different letters in the same column indicate significant differences.

## Results and discussion

### Generation of a high-yielding monacolin K mutant strain

**Figure 1:**
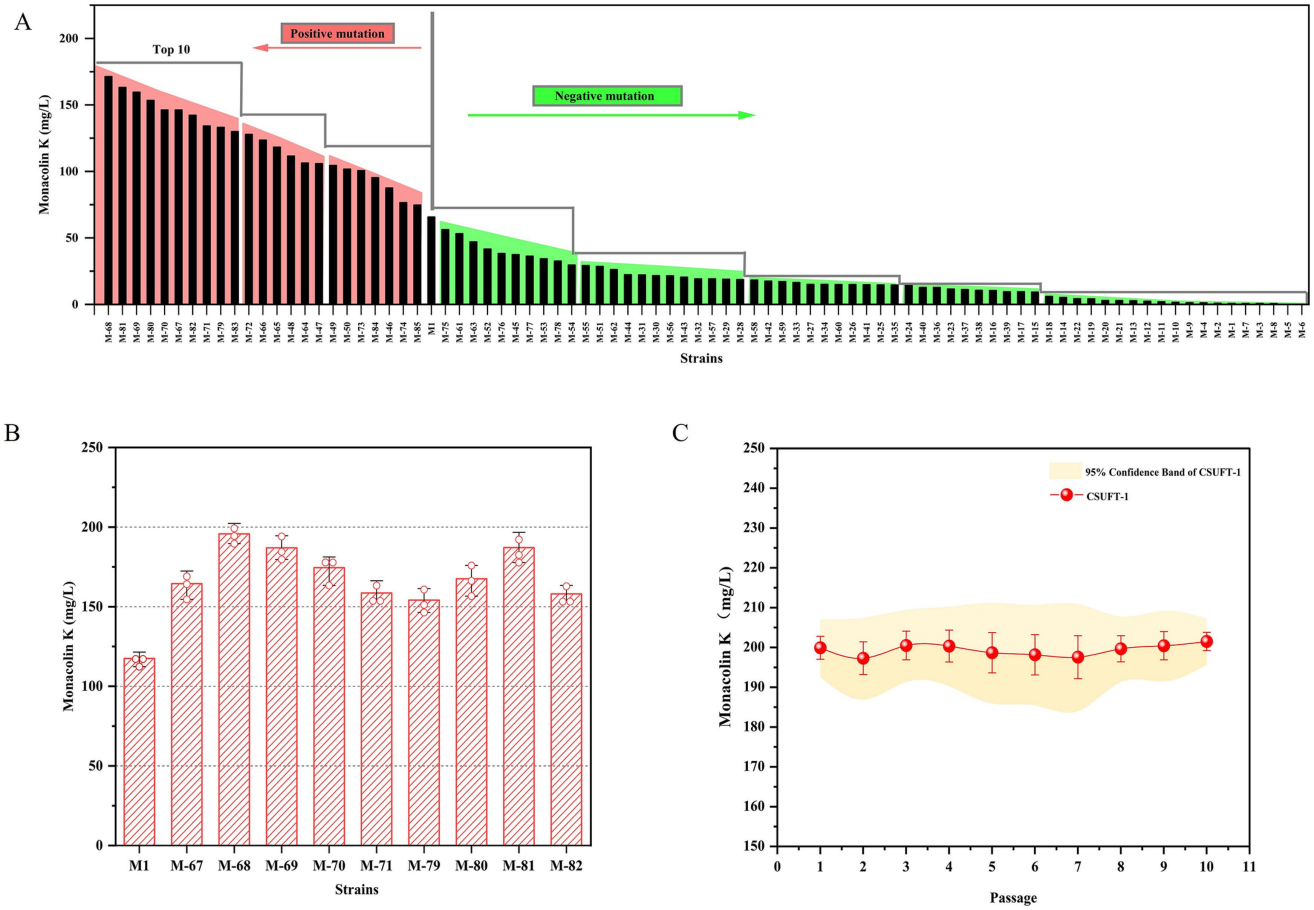
Generation of a high-yielding MK mutant strain. **(A)** The production of MK through 24-well fermentation by mutant strains. **(B)** The MK production of the rescreened mutants during submerged state fermentation in 250 mL Erlenmeyer flasks. **(C)** The MK production in submerged state fermentation by *M. purpureus* CSUFT-1 in ten successive batches.

LSF was prioritized over SSF due to its shorter cycle time and higher process efficiency, enabling rapid screening of mutant strains (Liu et al, 2019b). As shown in Figure 1, dual mutagenesis of the parental strain M1 mediated by ARTP and HIRFL irradiation, 85 mutant strains were selected for high-throughput screening through 24-well fermentation system. Among them, 35 mutant strains showed an increase in the production of MK, while 50 mutant strains exhibited a decrease (Figure 1A). Therefore, the top 10 mutant strains with enhanced MK production were identified, and they were used for further screening of the target in a 250 mL conical flask fermentation. The MK production of the strains (M-67, M-68, M-69, M-70, M-71, M-79, M-80, M-81, M-82, M-83) was 1.27- to 1.67-times higher than that of the parental strain M1 (Figure 1B). It is worth noting that among these mutant strains, strain M-68 exhibited the highest MK production, reaching 200 ± 2.5 mg/L. In addition, the MK production of the mutant strain M-68 remained comparable stability among generations, with a coefficient of variation value below 5% through 10 consecutive passages (Figure 1C). The effectiveness of double mutagenesis in targeted screening for beneficial traits has been verified. The mutant strains exhibit significant improvements in productivity and stability, providing a solid foundation for downstream applications (Bao et al., 2019; Hussien et al., 2021). Thus, the mutant strain M-68 with high-yielding MK was selected for further study.

### Growth characteristics analysis of the mutant strain

**Figure 2:**
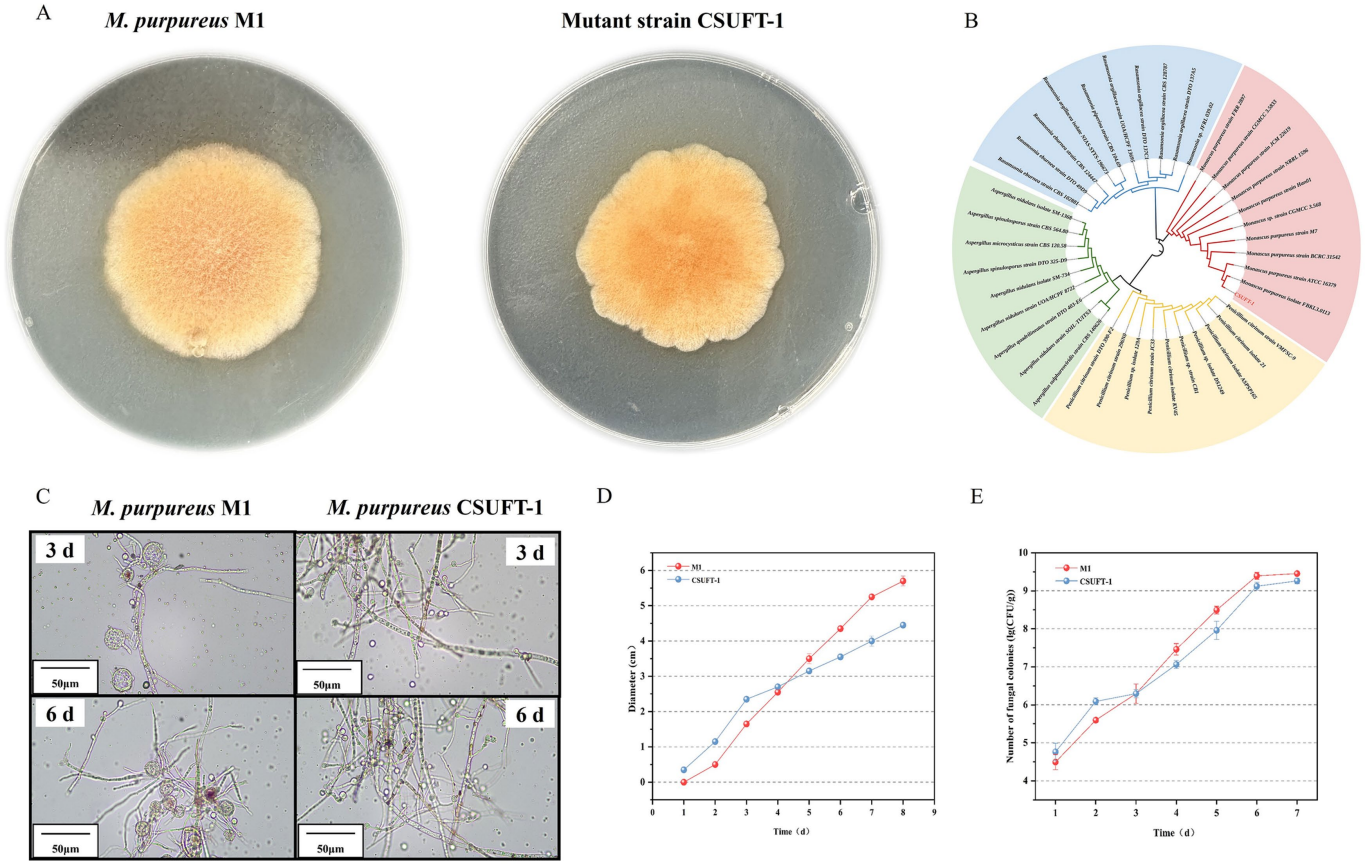
**(A)** The colony morphology of *M. purpureus* M1 and CSUFT-1 on the PDA medium. **(B)** Evolutionary tree of the ITS sequence of mutant strain CSUFT-1. **(C)** Mycelial morphology of *M. purpureus* M1 and CSUFT-1. **(D)** Colony diameter changes of *M. purpureus* M1 and CSUFT-1 on PDA medium. **(E)** Colony counts of *M. purpureus* M1 and CSUFT-1 during solid state fermentation using indica rice as the substrate.

In the present study, the mutant strain M68 exhibited typical morphological characteristics of *Monascus* genus on PDA medium for 7 days. However, during the solid-state cultivation period, compared with the original strain *M. purpureus* M1, the mutant strain exhibited different morphology, specifically, the colonies appeared denser and more compact (Figure 2A). Through ITS fragment analysis and observation of colony morphology on PDA medium, this mutant strain was identified as *M. purpureus* (Figure 2B), and was named *M. purpureus* CSUFT-1, stored in CCTCC with the number of 20,241,399. Besides, the abundance of cleistothecia within the mycelium indicates that the original strain predominantly utilizes sexual reproduction as its primary reproductive mode, whereas *M. purpureus* CSUFT-1 displayed a higher number of asexually produced conidiospore (Figure 2C). As a cholesterol synthesis inhibitor, MK may indirectly modulate spore release or germination through alterations in cell membrane fluidity (Huang et al., 2013). The conidiospore formation in *M. purpureus* CSUFT-1 is likely coupled with high production of MK. The results show that mutations in endogenous genes can induce changes in growth characteristics and increase the production of MK.

Although the colony diameter of the original strain was predominant during the later stage of growth on PDA medium, the mutant strain demonstrated a more rapid radial expansion during the initial 4 days of incubation, as evidenced by its larger colony diameter compared to *M. purpureus* M1 (Figure 2D). This suggests that *M. purpureus* CSUFT-1 possess a competitive advantage in substrate colonization during the early phases of cultivation. Besides, a similar temporal growth pattern was observed during SSF process, *M. purpureus* CSUFT-1 exhibited superior reproductive capacity within the first 72 h in the *Monascus*-fermented rice substrate (Figure 2E), potentially due to its enhanced utilization of readily available carbon sources. In subsequent work, we will employ whole-genome sequencing, transcriptomic analysis, and gene knockout or overexpression strategies to dissect the phenomenon in detail. Furthermore, compared to the parental strain M1, the mutant exhibited a significant reduction in both colony count and diameter upon completion of the cultivation process. These findings shows that mutant strain with rapid growth characteristics that do not extensively utilize carbon sources for biomass accumulation, represents ideal candidate for industrial-scale high-yield production of target metabolite.

### Screening optimal fermentation substrate for enhanced monacolin K production

The production of secondary metabolites during SSF is intricately linked to the fermentation substrate, as the substrate composition, physical structure, and physicochemical properties directly influence microbial metabolism and metabolic pathway activation (Srianta et al., 2016). In our previous study, indica rice was determined as the optimal carbon resource for RYR production through comparison of the activities of amylase and glucoamylase, index component content, mRNA level of genes involved in pigment biosynthesis in different rice varieties (Wang et al., 2023). Therefore, in this present study, indica rice was selected as the primary fermentation substrate; however, the effects of indica rice from different origins and supplementary components on MK biosynthesis during the SSF process remain unclear.

**Figure 3:**
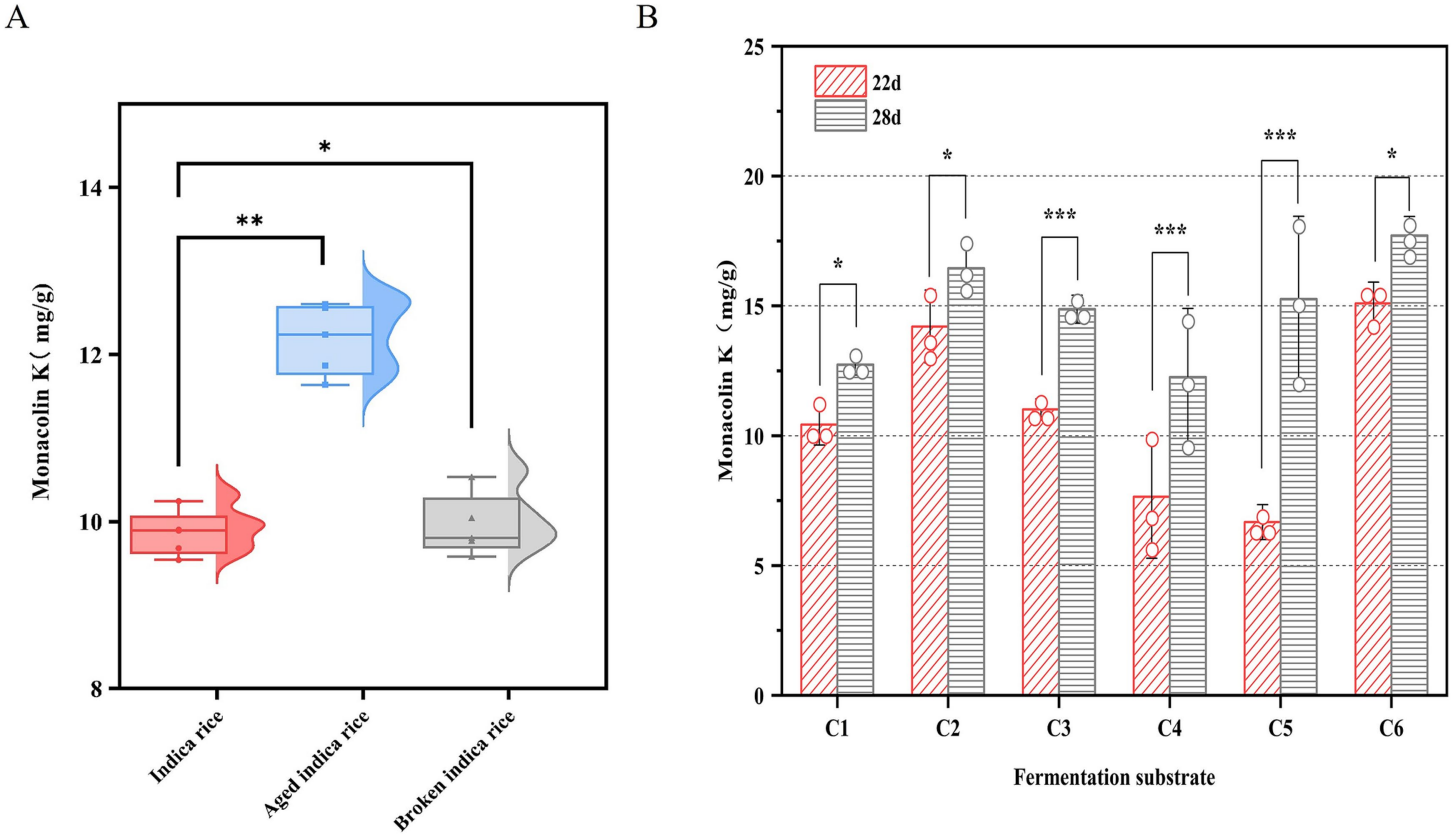
MK production of *M. purpureus* CSUFT-1 in various substrates. **(A)** Solid-state fermentation was conducted using indica rice, aged indica rice, and broken indica rice as substrates. **(B)** C1: Indica rice: Water (pH 4) = 50:5 (w/w); C2: Indica rice: Oat: Water (pH 4) = 50:5:5 (w/w); C3: Broken indica rice: Water (pH 4) = 50:5 (w/w); C4: Broken indica rice: Soybean powder: Wheat bran: Water (pH 4) = 50:1:1:10 (w/w); C5: Broken indica rice: Soybean powder: Wheat bran: Oat: Water (pH 4) = 50:1:2:1:1:10 (w/w); C6: Aged indica rice: Soybean powder: Wheat bran: Water (pH 4) = 50:1:1:10 (w/w). *p < 0.05, **p < 0.01, ***p < 0.001.

As shown in Figure 3A, after 22 days of fermentation, the MK content of aged indica rice (12.67 mg/g) was significantly higher than that of whole or broken indica rice. It has been reported that the amylose content of rice starch significantly increases, while the amylopectin content relatively decreases during the aging process (Hu et al., 2022). Amylose is released more slowly during fermentation, providing a more stable carbon source for microorganisms (Bui et al., 2020). Due to the long fermentation period, aged rice maintains a more stable carbon source for the sustained fermentation of *M. purpureus* CSUFT-1, thereby promoting MK accumulation.

To investigate the effects of components on MK production, the different fermentation substrates C1-C6 were used for SSF of *M. purpureus* CSUFT-1, with sampling conducted on day 22 and 28, respectively (Figure 3B). Results demonstrated the following MK content in RYR on day 28: 12.74 mg/g, 16.45 mg/g, 14.87 mg/g, 12.26 mg/g, 15.24 mg/g, and 18.05 mg/g in C1–C6, respectively, with C6 exhibiting the highest value. The damaged starch within it readily increase the accessible surface area for enzymatic action, breaks down complex carbohydrates into fermentable sugars, thereby providing a more readily utilizable carbon source (Rane et al., 2023). In this study, there were significant (*p* < 0.05) differences in MK content between day 22 and 28 in C1, C2, and C6, while extremely significant (*p* < 0.001) differences were observed in C3, C4, and C5, suggesting the suitability of C1, C2, and C6 as fermentation substrates for appropriately shortened-term fermentation processes. Besides, broken indica rice served as the sole carbon source in the C3, C4, and C5 fermentation substrate, its starch may have undergone structural changes, making it more susceptible to hydrolysis by enzymes produced by *M. purpureus* CSFUT-1 in the SSF later stage. Consequently, when the fermentation time was extended to 28 days, MK production showed a significant increase. Fermentation medium C2 exhibited a MK yield increase of 3.71 mg/g compared to C1, indicating oat supplementation enhances MK production. It reported that oats can be broken down into simpler metabolites during fermentation, this decomposition enhances the bioavailability of vitamins, minerals, and other nutrients while prolonging microbial metabolic activity (Djorgbenoo et al., 2023).

In SSF system, the carbon-release kinetics of substrates critically influence secondary metabolite biosynthesis. Moreover, nitrogen sources play a key regulatory role in MK biosynthesis (Srianta et al., 2016; Divate et al., 2017). Fermentation of *M. pilosus* MS-1 was performed by supplementing the substrate with 40% (w/w) soybean flour, yielding an MK production of 18.733 mg/g, representing a 33-fold increase compared to the control (Feng et al., 2014). The crude fiber in bran forms a porous framework, solving the caking issue of rice and soybean flour and increasing the oxygen diffusion rate by 2–3 times (Canedo et al., 2021). Fermentation substrates C6 containing soybean powder and wheat bran with a relatively appropriate proportion, significantly stimulated MK biosynthesis, demonstrating the most superior performance during the SSF of *M. purpureus* CSUFT-1.

### Optimization of substrate components for enhanced monacolin K production

**Figure 4:**
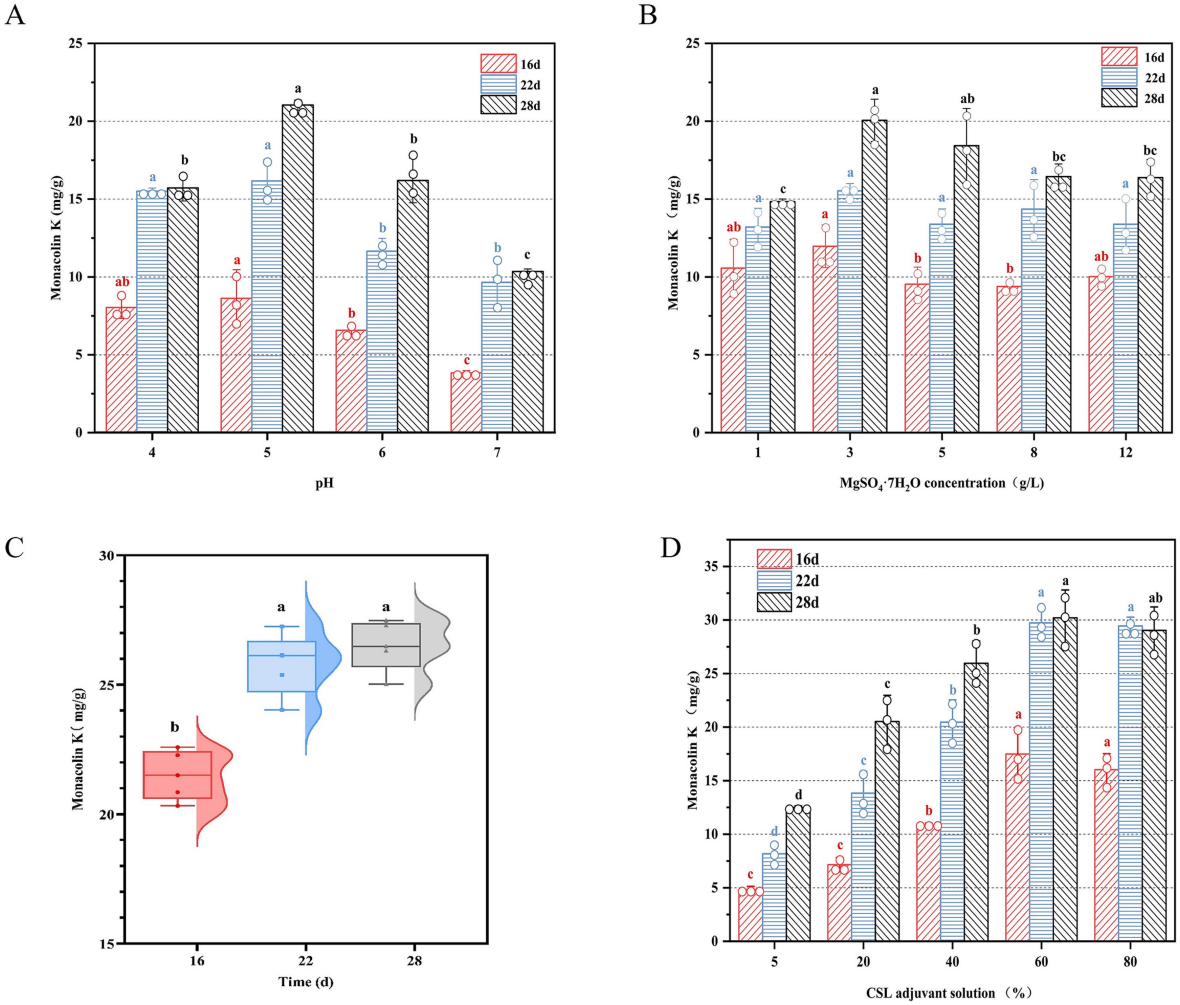
**(A)** The effect of pH value, **(B)** the concentration of MgSO₄·7H₂O, **(C)** 30% (v/v) CSL adjuvant solution, and the different concentration of CSL in the adjuvant solution **(D)** on the MK production during solid state fermentation by *M. purpureus* CSUFT-1.

To further enhance the MK content in RYR, the components of an adjuvant solution, which serves as a substitute for pure water with pH 4, have been optimized. It has been reported that the initial pH of culture medium exerts a significant regulatory effect on the proliferation rate and metabolic activity of fungi (Zhang et al., 2025). Thus, the effect of pH value in the basal pure water on the MK production was firstly evaluated. As illustrated in Figure 4A, MK production under pH 5 progressively increased to a peak of 21.42 mg/g with an increase of 6.19 mg/g during the 28 days fermentation period, whereas elevated pH resulted in a marked yield decline. It reveals that the optimal pH condition can significantly promotes MK biosynthesis. Likewise, the MK and citrinin productions were dramatically regulated through controlling the pH value during LSF (Lee et al., 2007). Besides, the initial pH value of the fermentation medium distinctly exhibits the regulating effect of lovastatin (also called MK) biosynthesis (Bizukojc et al., 2012). Environmental pH, as a primary control factor to microorganism, has multiple type of regulation to microbial metabolisms, including redox reaction and respiration (Jin and Kirk, 2018). Specifically, it governs bacterial proliferation and metabolic activity while shaping their biochemical profiles (Razmi et al., 2023). Therefore, precise control of fermentation system pH value is a key process parameter to achieve efficient MK production.

Mg^2+^ can promote the biomass, polysaccharide production, and secondary metabolite synthesis of fungi and act as an enzyme cofactor to regulate the biosynthesis and metabolism of *Monascus* (Lee et al., 2011). As depicted in Figure 4B, MK production in *Monascus*-fermented rice exhibited time- and concentration-dependent variations. With the progression of fermentation time, the yield of MK gradually increased. When the concentration of MgSO_4_·7H₂O in the basal pure water was added to 3 g/L, the yield of MK reached 20.06 mg/g on day 28. Mg^2+^ enters the cell through active transport and endocytosis, further stimulating pathways related to energy metabolism, such as glycolysis, the citric acid cycle, and the pentose phosphate pathway. However, high concentrations of Mg^2+^ may compete with other cations within the cell, inhibiting the activity of key enzymes and thereby affecting the production of secondary metabolites (Zhu et al., 2025). Therefore, precise modulation of MgSO_4_ supplementation level serves as a critical process parameter for balancing the mycelia growth and MK biosynthesis.

In microbial fermentation processes, adjuvant solutions are primarily added to optimize fermentation conditions and address limitations of the basal medium. These auxiliary solutions promote the growth of target microorganisms and maintain fermentation environment stability (Singh et al., 2017). Corn steep liquor (CSL), serving as a cost-effective fermentation medium rich in nitrogen sources, amino acids, vitamins, and minerals, significantly enhances microbial metabolic activity, demonstrates substantial application potential (Wahjudi et al., 2023). Figure 4C illustrates that substituting pure water with 30% (v/v) CSL adjuvant solution (CSLAS) with the optimized values of pH 5, 3 g/L of MgSO_4_·7H₂O, and 1.5 g/L of KH_2_PO_4_ in C6 significantly elevated MK content in RYR to 26.36 mg/g on day 28 (an increase of 24%). Compared to pure water, CSL provides “all-nutrient” support, reducing the need for additional chemically synthesized nutrients, thereby enhancing fermentation efficiency while lowering production costs (Zhu et al., 2019). MK biosynthesis efficiency by *M. purpureus* CSUFT-1 during SSF exhibited a gradual increase with fermentation time when CSLAS concentration was elevated from 5 to 60%, peaking at 32.71 mg/g of MK on day 28 when addition of 60% CSLAS with the injection volume of 20% (v/w) in C6 (Figure 4D). Similarly, during the fermentation of *Sporidiobolus pararoseus* JD-2, supplementation with 20 g/L of CSL enhanced microbial oil and exopolysaccharide yields by 8.7 and 12.9%, respectively (Guo et al., 2022). *Arthrospira platensis* yielded the maximal fibrinolytic activity of 268.14 U/mg under mixotrophic conditions with 0.2% (v/v) CSL supplementation in liquid medium (de Barros et al., 2020).

### Scale-up production of red yeast rice enriched with monacolin K

**Figure 5:**
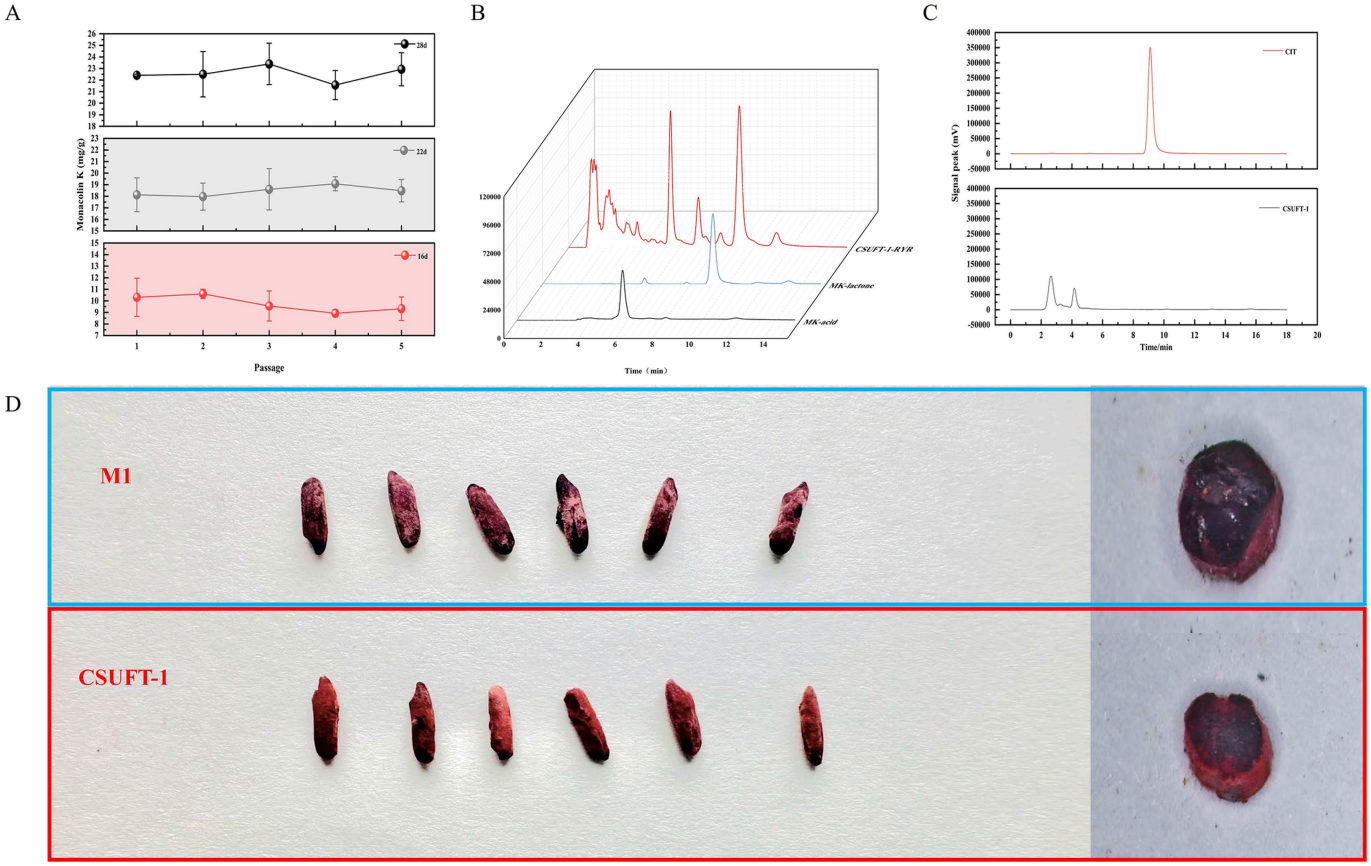
**(A)** Monacolin K levels in red yeast rice were monitored over five successive batches of scaled-up fermentation by *M. purpureus* CSUFT-1 on day 16, 22, and 28. **(B)** The quantification of MK in solid-state fermented red yeast rice by using high-performance liquid chromatography (HPLC). **(C)** The quantification of citrinin levels in liquid fermentation using HPLC. **(D)** Surface morphology and cross-sectional microstructure in red yeast rice produced by *M. purpureus* M1 and CSUFT-1.

Process scale-up is pivotal for translating laboratory-scale fermentation into industrial production (Tulsyan, 2023). Scale-up fermentation was implemented using the optimized C6 formulation, achieving a 7-fold amplification (from 30 g to 210 g) by using the ampliative wide-mouth glass bottles (46 × 175 × 80 mm). Figure 5A shows that MK yield stabilized within in a range of 8 to 11 mg/g on day 16, with <10% variability across five subcultures; subsequently, by day 22, it increased to 17–20 mg/g, and further achieved to a maximal production of 24.66 mg/g on day 28, demonstrating the high consistency in MK content across RYR produced during SSF by *M. purpureus* CSUFT-1. What’s more, the detected peaks of HPLC are illustrated in Figure 5B, displaying an acid-to-lactone type MK ratio of 0.66. Additionally, citrinin was not detected in bio-manufactured RYR using HPLC method (Figure 5C). Observations revealed the macroscopic features of RYR produced by the original strain *M. purpureus* M1 and the mutant strain CSUFT-1 (Figure 5D). Samples from the *M. purpureus* M1 group exhibited a purplish-red to deep red coloration, while those from *M. purpureus* CSUFT-1 group displayed a lighter red tone. Additionally, RYR samples from the control group displayed wrinkled surface texture, whereas *M. purpureus* CSUFT-1 group exhibited a comparatively smooth and plump surface morphology. In addition, the cross-sectional analysis of RYR shows that the complete utilization of the two types of substrates results in no residual unfermented starch particles in the solid-state fermentation substrate. These results demonstrate that optimized critical components effectively balanced the cell growth with secondary metabolism during scale-up fermentation.

### Mechanistic analysis of MK overproduction based on gene cluster expression

**Figure 6:**
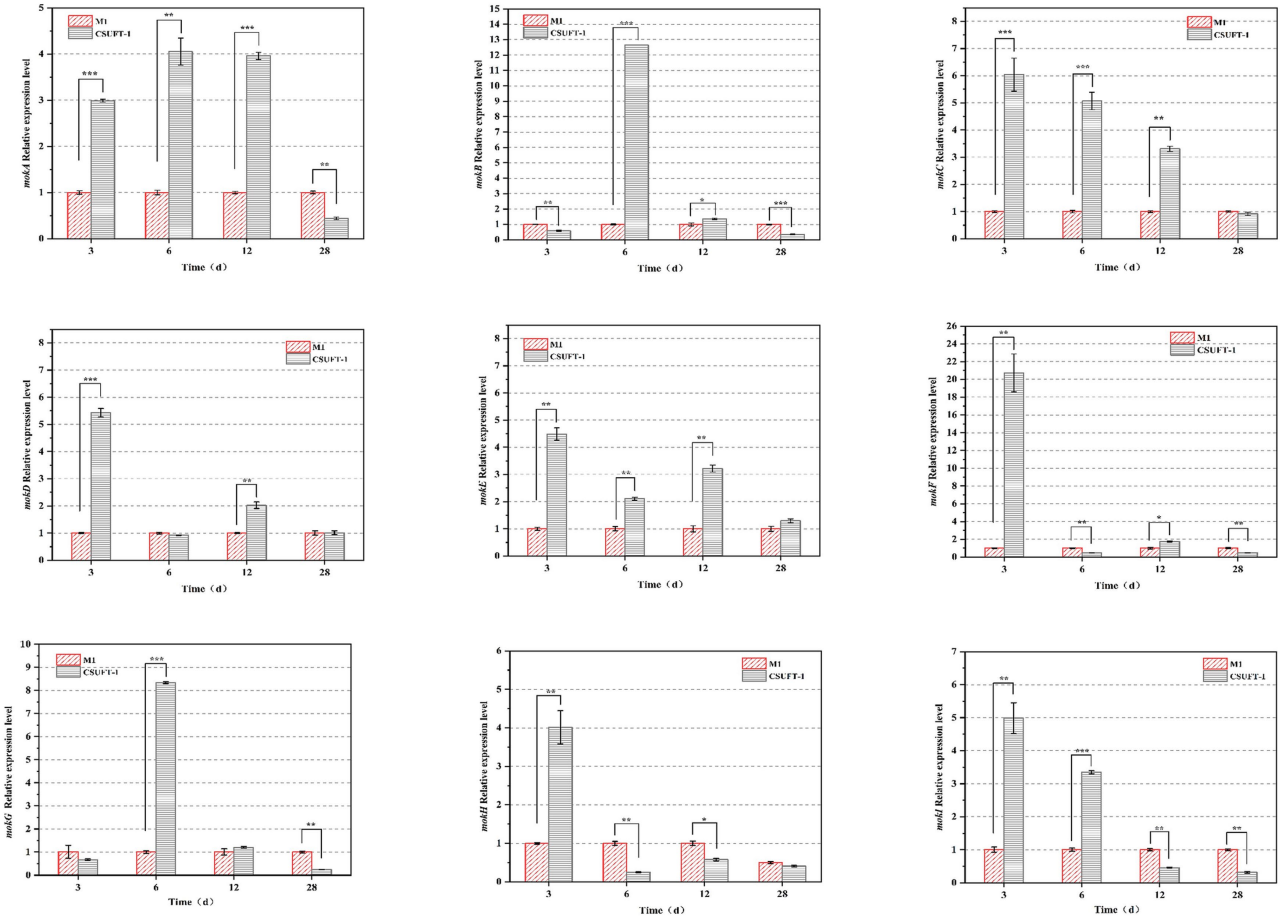
The mRNA levels of the genes located in the MK biosynthetic gene cluster. *p < 0.05, **p < 0.01, ***p < 0.001.

RT-qPCR analysis revealed significant temporal differences in the expression patterns of the MK biosynthetic gene cluster (*mo*k*A–I*) between *M. purpureus* CSUFT-1 and M1 during SSF process (Figure 6). Specifically, during the early fermentation stage (3 d), the mRNA levels of seven genes (*mokA*, *mokC*, *mokD*, *mokE*, *mokF*, *mokH*, *mokI*) in *M. purpureus* CSUFT-1 were markedly higher than those in *M. purpureus* M1. Similarly, overexpression of the *mokI* gene in *M. pilosus* significantly enhanced MK production, with this gene showing the most substantial upregulation during early fermentation stage (Huang et al., 2024). Besides, *mokC* and *mokF* exhibited 6.03-fold and 22-fold upregulation, *mokC* directly participates in backbone synthesis rather than transcriptional regulation. In engineered strains overexpressing *mokC*, MK production reaches 230% of that in the parental strain, demonstrating a strong positive correlation between its expression level and MK yield (Zhang et al., 2019). Meanwhile, upregulation of *mokF* facilitates efficient metabolic flux conversion toward the target bioactive product. This indicates activation of the entire gene cluster dramatically stimulate the MK biosynthesis. On day 6 of cultivation, the expression of *mokB* (encoding polyketide synthase) in *M. purpureus* CSUFT-1 was significantly up-regulated by 12.39-fold, which drives the biosynthesis of the diketone side chain in MK. In addition, compared to the control group, the expressions of *mokA*, *mokC*, and *mokE* in the mutant strain exhibited significant upregulations of 3.96-, 3.31-, and 3.21-fold in SSF process on day 12, respectively. It signifies coordinated activation of MK biosynthetic cluster, directly driving enhanced precursor flux and structural assembly of this polyketide (Zhang et al., 2020). In addition, the sustained overexpression of *mokE* in *M. purpureus* CSUFT-1 during SSF suggests its pivotal role as a pathway-specific regulatory protein. *mokE* can compensate for the functional loss of the enoyl reductase domain in *mokA* caused by active-site amino acid deletion, thereby maintaining MK biosynthesis (Yao et al., 2025). The results showed that a large proportion of genes in the cluster exhibit downregulated mRNA level at the terminal phase of SSF (28th day). Feedback inhibition or substrate competition mechanisms triggered by transient early gene overexpression, which suppressed later transcriptional activity (Wang et al., 2017).

**Figure 7:**
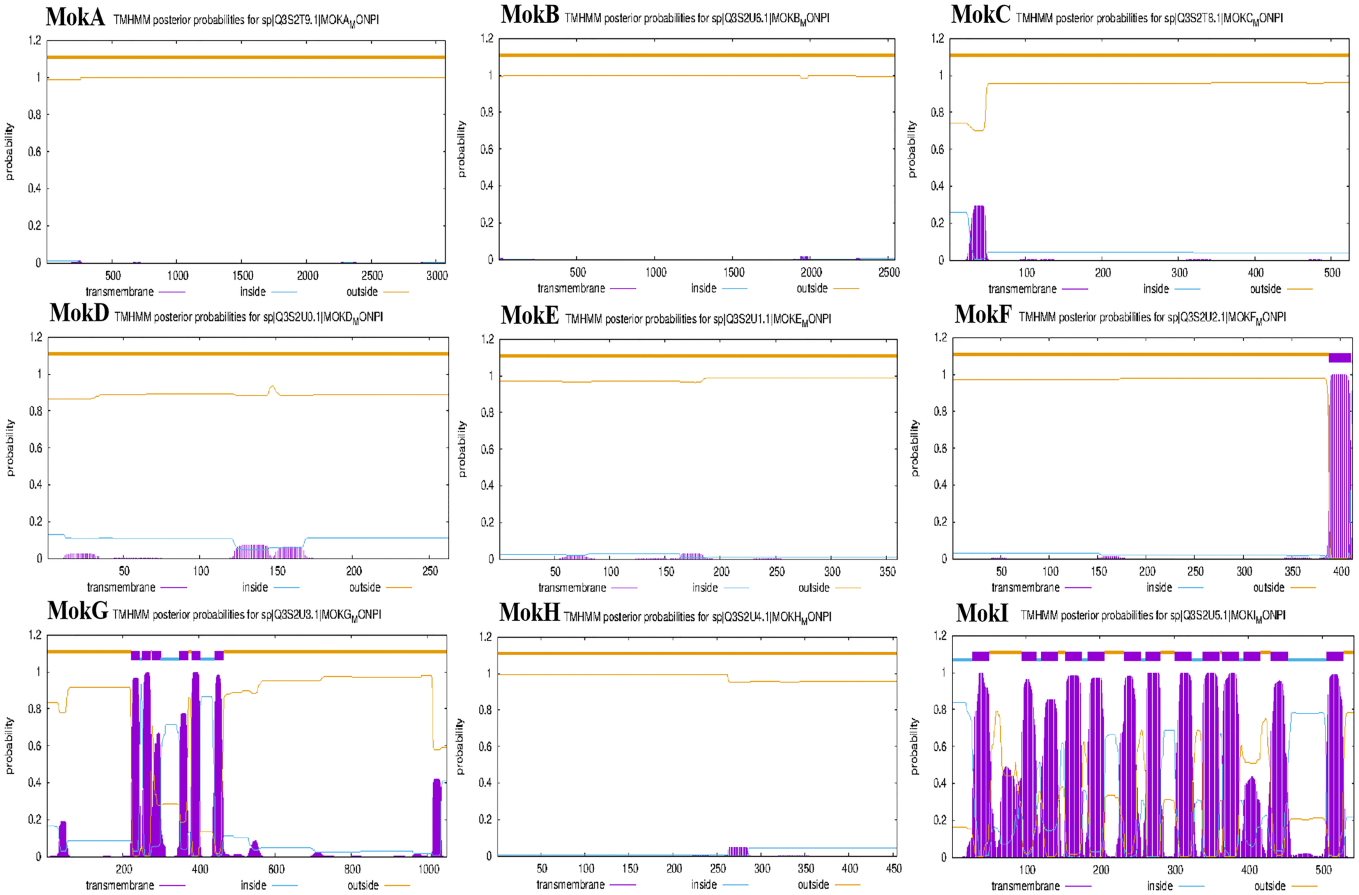
The transmembrane structure analysis of proteins (MokA-MokI) by TMHMM Server v. 2.0.

**Figure 8:**
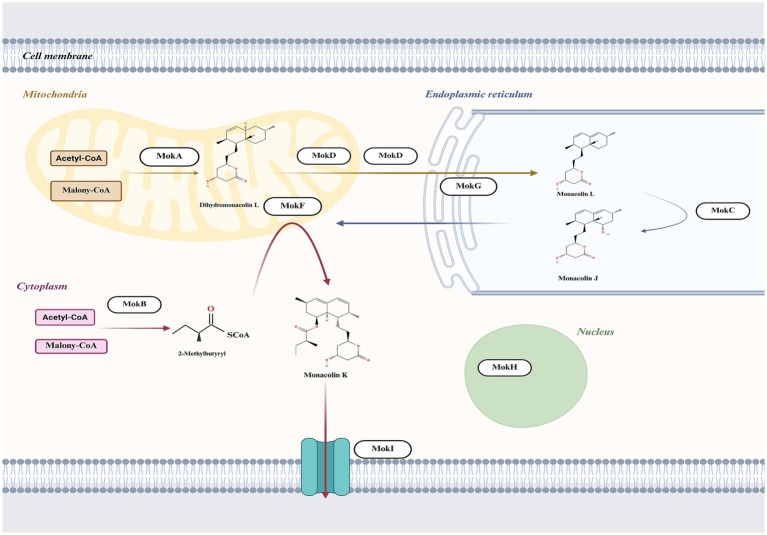
The proposed intracellular biosynthesis pathway of MK in *M. purpureus* based on the subcellular localization predictions of proteins situated within the MK biosynthetic gene cluster.

The subcellular localization is crucial for understanding the protein’s role in cellular processes, as it determines how the protein interacts with other cellular components and contributes to specific biological activities (Gillani and Pollastri, 2024). Thus, we integrated protein-predicted subcellular localization with the MK biosynthetic pathway to explicitly exhibit the efficient biosynthesis of MK in *M. purpureus* CSUFT-1. Protein localization predictions (Table 3) and transmembrane structure analyses (Figure 7) reveal that MokB (polyketide synthase) and MokE (dehydrogenase) are cytoplasmic, whereas MokA (polyketide synthase), MokD (oxidoreductase), and MokF (transesterase) localize to the mitochondria. MokC (P450 monooxygenase) and MokG (HMG-CoA reductase has 6 predicted transmembrane helices) are predicted in the endoplasmic reticulum, MokH (transcription factor) in the nucleus, and MokI (efflux pump with 13 predicted transmembrane helices) in the cytoplasmic membrane. Experimental results demonstrated that *mokA*, *mokC*, *mokD*, *mokF*, and *mokI* were synergistically upregulated during the early fermentation phase. This promoted carbon backbone synthesis (by MokA, C), catalyzed intermediate conversion (by MokD, F), and relieved feedback inhibition (by MokI), collectively enabling efficient MK biosynthesis while preventing intermediate accumulation (Figure 8). Synchronous upregulation of *mokB* and *mokG* accelerated the core stereochemical assembly stage of MK synthesis. Persistent upregulation of *mokE* redirected metabolic flux toward the MK synthesis pathway, thereby enhancing yield. Regulation of these genes through genetic engineering approaches can significantly enhance MK biosynthesis efficiency (Dai et al., 2021; Gong et al., 2024). For instance, Adding 512 μM linoleic acid can upregulate the transcription level of the *mokA* gene, resulting in a 1.35-fold increase in MK production (Huang et al., 2018). Knockout of the *mokF* gene causes obstruction of specific steps in the metabolic pathway, thereby reducing the MK biosynthetic efficiency (Zhang et al., 2022). Besides, compared with the wild-type strain, the MK production of the overexpressed strains *mokC*, *mokD*, *mokE* and *mokI* increased by 234.3, 220.8, 89.5 and 10%, respectively, (Zhang et al., 2019). These findings imply the significance of these genes and their differential regulatory effects on the MK biosynthetic pathway.

**Table 3:** Predicted subcellular localizations of proteins encoded by the monacolin K biosynthetic gene cluster.

Protein	Function	Localization
MokA	Polyketide synthase	Mitochondria
MokB	Polyketide synthase	Cytoplasm
MokC	P450 monooxygenase	Endoplasmic reticulum
MokD	Oxidoreductase	Mitochondria
MokE	Dehydrogenase	Cytoplasm
MokF	Translipase	Mitochondria
MokG	HMG-CoA reductase	Endoplasmic reticulum
MokH	Transcription factor	Cell nucleus
MokI	Efflux pump	Cytomembrane

## Conclusion

This study employed ARTP-HIRFL composite mutagenesis to isolate a high yield *Monascus* mutant strain *M. purpureus* CSUFT-1, achieving a 1.67-fold increase in MK yield compared to the parental strain. Through systematic optimization of SSF parameters, MK content in RYR was enhanced to 32.71 mg/g. Additionally, it maintained high consistency over five successive generations in the scale-up production, with a stable yield of 20.68–24.66 mg/g, confirming the industrial robustness of the mutant strain. RT-qPCR analysis revealed upregulation of seven critical genes (e.g., *mokA, mokC*, and *mokF*) in MK biosynthesis during the early stage of fermentation, the sustained overexpression of *mokE* during SSF in the mutant strain compared to the parental strain suggests its pivotal role. Our findings illustrate that the mutagenesis-driven optimization of temporal gene expression patterns in *M. purpureus* CSUFT-1 significantly enhanced MK accumulation efficiency. The subcellular localization and temporal expression dynamics of the proteins located in the MK biosynthetic gene cluster offer valuable insights into the molecular mechanisms underlying MK synthesis and regulation, thereby precisely contributing to the high-efficiency and safe industrial production of RYR through using biotechnological applications.

## Data Availability

The original contributions presented in the study are included in the article/supplementary material, further inquiries can be directed to the corresponding authors.

## References

[ref1] Bai J. Gong Z. Shu M. Zhao H. Ye F. Tang C.. (2022). Increased water-soluble yellow *Monascus* pigment productivity via dual mutagenesis and submerged repeated-batch fermentation of *Monascus purpureus*. Front. Microbiol. 13:914828. doi: 10.3389/fmicb.2022.914828, PMID: 35756045 PMC9218666

[ref2] Bao H. X. Zhang X. Su H. Z. Li L. Y. Lv Z. Z. Zhang X. Y. (2019). Study on the hydrogen production ability of high-efficiency bacteria and synergistic fermentation of maize straw by a combination of strains. RSC Adv. 9, 9030–9040. doi: 10.1039/c9ra00165d, PMID: 35517707 PMC9062066

[ref3] Bizukojc M. Pawlak M. Boruta T. Gonciarz J. (2012). Effect of pH on biosynthesis of lovastatin and other secondary metabolites by *aspergillus terreus* ATCC 20542. J. Biotechnol. 162, 253–261. doi: 10.1016/j.jbiotec.2012.09.007, PMID: 22995742

[ref4] Bui A. T. Williams B. A. Hoedt E. C. Morrison M. Mikkelsen D. Gidley M. J. (2020). High amylose wheat starch structures display unique fermentability characteristics, microbial community shifts and enzyme degradation profiles. Food Funct. 11, 5635–5646. doi: 10.1039/d0fo00198h, PMID: 32537617

[ref5] Canedo M. S. Figueiredo M. F. S. Thomik M. Vorhauer-Huget N. Tsotsas E. Thoméo J. C. (2021). Porosity and pore size distribution of beds composed by sugarcane bagasse and wheat bran for solid-state cultivation. Powder Technol. 386, 166–175. doi: 10.1016/j.powtec.2021.03.039

[ref6] Chai X. Ai Z. Liu J. Guo T. Wu J. Bai J.. (2020). Effects of pigment and citrinin biosynthesis on the metabolism and morphology of *Monascus purpureus* in submerged fermentation. Food Sci. Biotechnol. 29, 927–937. doi: 10.1007/s10068-020-00745-3, PMID: 32582455 PMC7297905

[ref7] Chen Y. P. Tseng C. P. Liaw L. L. Wang C. L. Chen I. C. Wu W. J.. (2008). Cloning and characterization of monacolin K biosynthetic gene cluster from *Monascus pilosus*. J. Agric. Food Chem. 56, 5639–5646. doi: 10.1021/jf800595k, PMID: 18578535

[ref8] Dai W. Shao Y. Chen F. (2021). Production of monacolin K in *Monascus pilosus*: comparison between industrial strains and analysis of its gene clusters. Microorganisms 9:747. doi: 10.3390/microorganisms9040747, PMID: 33918292 PMC8065618

[ref9] de Barros P. D. S. Silva P. E. C. E. Nascimento T. P. Costa R. M. P. B. Bezerra R. P. Porto A. L. F. (2020). Fibrinolytic enzyme from *Arthrospira platensis* cultivated in medium culture supplemented with corn steep liquor. Int. J. Biol. Macromol. 164, 3446–3453. doi: 10.1016/j.ijbiomac.2020.08.217, PMID: 32882274

[ref10] Divate R. D. Wang C.-C. Chou S.-T. Chang C.-T. Wang P.-M. Chung Y.-C. (2017). Using wheat bran and soybean meal as solid state fermentation substances for the production of Xylaria nigripes with bioactivities. J. Taiwan Inst. Chem. Eng. 70, 127–133. doi: 10.1016/j.jtice.2016.11.003

[ref11] Djorgbenoo R. Hu J. Hu C. Sang S. (2023). Fermented oats as a novel functional food. Nutrients 15:3521. doi: 10.3390/nu15163521, PMID: 37630712 PMC10459665

[ref12] Feng Y. Shao Y. Zhou Y. Chen F. (2014). Production and optimization of monacolin K by citrinin-free *Monascus pilosus* MS-1 in solid-state fermentation using non-glutinous rice and soybean flours as substrate. Eur. Food Res. Technol. 239, 629–636. doi: 10.1007/s00217-014-2259-z

[ref9001] Gillani M. Pollastri G. (2024). Protein subcellular localization prediction tools. Computational and Structural Biotechnology Journal 23, 1796–1807. doi: 10.1016/j.csbj.2024.04.032 38707539 PMC11066471

[ref13] Gong Y. Li S. Zhao D. Yuan X. Zhou Y. Chen F.. (2024). From random perturbation to precise targeting: a comprehensive review of methods for studying gene function in *Monascus* species. J. Fungi 10:892. doi: 10.3390/jof10120892, PMID: 39728388 PMC11678829

[ref14] Guo Y.-F. Wang M.-Q. Wang Y.-L. Wang H.-T. Xu J.-Z. (2022). Controlling the formation of foams in broth to promote the co-production of microbial oil and exopolysaccharide in fed-batch fermentation. Fermentation 8:68. doi: 10.3390/fermentation8020068

[ref15] Hu H. Li S. Pan D. Wang K. Qiu M. Qiu Z.. (2022). The variation of Rice quality and relevant starch structure during Long-term storage. Agriculture 12:1211. doi: 10.3390/agriculture12081211

[ref16] Huang J. Liao N. Li H. (2018). Linoleic acid enhance the production of moncolin K and red pigments in *Monascus ruber* by activating *mokH* and *mokA*, and by accelerating cAMP-PkA pathway. Int. J. Biol. Macromol. 109, 950–954. doi: 10.1016/j.ijbiomac.2017.11.074, PMID: 29162465

[ref17] Huang C.-H. Shiu S.-M. Wu M.-T. Chen W.-L. Wang S.-G. Lee H.-M. (2013). Monacolin K affects lipid metabolism through SIRT1/AMPK pathway in HepG2 cells. Arch. Pharm. Res. 36, 1541–1551. doi: 10.1007/s12272-013-0150-2, PMID: 23657807

[ref18] Huang Z. Xiao L. Mo W. Zhang Y. Cai Y. Huang S.. (2024). Molecular mechanism of *Mok I* gene overexpression in enhancing monacolin K production in *Monascus pilosus*. J. Fungi (Basel) 10:721. doi: 10.3390/jof10100721, PMID: 39452673 PMC11508744

[ref19] Hussien R. H. M. Ezzat S. M. El Sheikh A. A. Taylor J. W. D. Butt T. M. (2021). Comparative study of fungal stability between *Metarhizium* strains after successive subculture. Egypt. J. Biol. Pest Control 31:2. doi: 10.1186/s41938-020-00348-4

[ref20] Jin Q. Kirk M. F. (2018). pH as a primary control in environmental microbiology: 1. Thermodynamic perspective. Front. Environ. Sci. 6:21. doi: 10.3389/fenvs.2018.00021

[ref21] Kim D. Ku S. (2018). Beneficial effects of *Monascus* sp. KCCM 10093 pigments and derivatives: a Mini review. Molecules 23:98. doi: 10.3390/molecules23010098, PMID: 29301350 PMC6017178

[ref22] Lee C.-L. Hung H.-K. Wang J.-J. Pan T.-M. (2007). Improving the ratio of monacolin K to Citrinin production of *Monascus purpureus* NTU 568 under Dioscorea Medium through the mediation of pH value and ethanol addition. J. Agric. Food Chem. 55, 6493–6502. doi: 10.1021/jf0711946, PMID: 17636932

[ref23] Lee C.-L. Kung Y.-H. Wang J.-J. Lung T.-Y. Pan T.-M. (2011). Enhanced Hypolipidemic effect and safety of red Mold Dioscorea cultured in Deep Ocean water. J. Agric. Food Chem. 59, 8199–8207. doi: 10.1021/jf201948v, PMID: 21732592

[ref24] Lee C.-L. Wang J.-J. Kuo S.-L. Pan T.-M. (2006). *Monascus* fermentation of dioscorea for increasing the production of cholesterol-lowering agent—monacolin K and antiinflammation agent—monascin. Appl. Microbiol. Biotechnol. 72, 1254–1262. doi: 10.1007/s00253-006-0404-8 16568313

[ref25] Li S.-W. Li M. Song H.-P. Feng J.-L. Tai X.-S. (2011). Induction of a high-yield lovastatin mutant of aspergillus terreus by ^12^C^6+^ heavy-ion beam irradiation and the influence of culture conditions on lovastatin production under submerged fermentation. Appl. Biochem. Biotechnol. 165, 913–925. doi: 10.1007/s12010-011-9308-x, PMID: 21710210

[ref26] Liu J. Chai X. Y. Guo T. Wu J. Y. Yang P. P. Luo Y. C.. (2019a). Disruption of the ergosterol biosynthetic pathway results in increased membrane permeability, causing overproduction and secretion of extracellular *Monascus* pigments in submerged fermentation. J. Agric. Food Chem. 67, 13673–13683. doi: 10.1021/acs.jafc.9b05872, PMID: 31617717

[ref27] Liu C. Cheng L. Yang M. He Z. Jia Y. Xu L.. (2025). Screening for safe and efficient Monascus strains with functions of lowering blood lipids, blood glucose, and blood pressure. Foods 14:835. doi: 10.3390/foods14050835, PMID: 40077539 PMC11899137

[ref28] Liu K. Fang H. Cui F. Nyabako B. A. Tao T. Zan X.. (2020). ARTP mutation and adaptive laboratory evolution improve probiotic performance of *Bacillus coagulans*. Appl. Microbiol. Biotechnol. 104, 6363–6373. doi: 10.1007/s00253-020-10703-y, PMID: 32474797

[ref29] Liu J. Guo T. Luo Y. Chai X. Wu J. Zhao W.. (2019b). Enhancement of *Monascus* pigment productivity via a simultaneous fermentation process and separation system using immobilized-cell fermentation. Bioresour. Technol. 272, 552–560. doi: 10.1016/j.biortech.2018.10.072, PMID: 30396112

[ref30] Liu X. Sun A. Li Q. Du Y. Zhao T. (2022). A systematic study of the production of monacolin K by solid state fermentation of *Monascus ruber*. AMB Express 12:29. doi: 10.1186/s13568-022-01368-z, PMID: 35239075 PMC8894543

[ref31] Rane D. V. Pawar P. P. Odaneth A. A. Lali A. M. (2023). Microbial oil production by the oleaginous red yeast, *Rhodotorula glutinis* NCIM 3168, using corncob hydrolysate. Biomass Convers. Biorefinery 13, 1987–1997. doi: 10.1007/s13399-021-01298-z

[ref32] Razmi N. Lazouskaya M. Pajcin I. Petrovic B. Grahovac J. Simic M.. (2023). Monitoring the effect of pH on the growth of pathogenic bacteria using electrical impedance spectroscopy. Results Eng. 20:101425. doi: 10.1016/j.rineng.2023.101425

[ref33] Singh V. Haque S. Niwas R. Srivastava A. Pasupuleti M. Tripathi C. K. M. (2017). Strategies for fermentation medium optimization: an in-depth review. Front. Microbiol. 7:2087. doi: 10.3389/fmicb.2016.02087, PMID: 28111566 PMC5216682

[ref34] Srianta I. Kusdiyantini E. Zubaidah E. Ristiarini S. Nugerahani I. Alvin A.. (2021). Utilization of agro-industrial by-products in *Monascus* fermentation: a review. Bioresour. Bioprocess. 8:129. doi: 10.1186/s40643-021-00473-4, PMID: 38650194 PMC10992953

[ref35] Srianta I. Zubaidah E. Estiasih T. Yamada M. Harijono (2016). Comparison of *Monascus purpureus* growth, pigment production and composition on different cereal substrates with solid state fermentation. Biocatal. Agric. Biotechnol. 7, 181–186. doi: 10.1016/j.bcab.2016.05.011

[ref36] Su Y.-C. Wang J.-J. Lin T.-T. Pan T.-M. (2003). Production of the secondary metabolites γ-aminobutyric acid and monacolin K by *Monascus*. J. Ind. Microbiol. Biotechnol. 30, 41–46. doi: 10.1007/s10295-002-0001-5, PMID: 12545385

[ref37] Sun J. Li J. Yao L. Zheng Y. Yuan J. Wang D. (2023). UV-ARTP-DES compound mutagenesis breeding improves natamycin production of Streptomyces natalensis HW-2 and reveals transcriptional changes by RNA-seq. Food Sci. Biotechnol. 32, 341–352. doi: 10.1007/s10068-022-01191-z, PMID: 36778090 PMC9905406

[ref38] Tulsyan A. (2023). Industrial batch monitoring for cell culture processes during process scale-ups: data challenges and solutions. IFAC-PapersOnLine 56, 570–575. doi: 10.1016/j.ifacol.2023.10.1628

[ref39] Wahjudi S. M. W. Petrzik T. Oudenne F. Lera Calvo C. Büchs J. (2023). Unraveling the potential and constraints associated with corn steep liquor as a nutrient source for industrial fermentations. Biotechnol. Prog. 39:e3386. doi: 10.1002/btpr.3386, PMID: 37634939

[ref40] Wang R. Burton J. L. Solomon M. J. (2017). Transcriptional and post-transcriptional regulation of Cdc20 during the spindle assembly checkpoint in *S. cerevisiae*. Cell. Signal. 33, 41–48. doi: 10.1016/j.cellsig.2017.02.003, PMID: 28189585 PMC5394925

[ref41] Wang Y. Ye F. Zhou B. Liang Y. Lin Q. Lu D.. (2023). Comparative analysis of different rice substrates for solid-state fermentation by a citrinin-free *Monascus purpureus* mutant strain with high pigment production. Food Biosci. 56:103245. doi: 10.1016/j.fbio.2023.103245

[ref42] Wen Q. Cao X. Chen Z. Xiong Z. Liu J. Cheng Z.. (2020). An overview of *Monascus* fermentation processes for monacolin K production. Open Chem. 18, 10–21. doi: 10.1515/chem-2020-0006

[ref43] Wu A. Li L. Zhang S. Lin Q. Liu J. (2023). Optimization of the hongqu starter preparation process for the manufacturing of red mold rice with high gamma-aminobutyric acid production by solid-state fermentation. Biotechnol. Appl. Biochem. 70, 458–468. doi: 10.1002/bab.2370, PMID: 35662255

[ref44] Xiong Z. Cao X. Wen Q. Chen Z. Cheng Z. Huang X.. (2019). An overview of the bioactivity of monacolin K / lovastatin. Food Chem. Toxicol. 131:110585. doi: 10.1016/j.fct.2019.110585, PMID: 31207306

[ref45] Yang M. Yu Y. Zhai H. Sheng J. Xing T. Chen G. (2018). Ecological dyeing of silk fabric with *Monascus*. J. Text. Inst. 109, 1329–1334. doi: 10.1080/00405000.2018.1423932

[ref46] Yao T. Wang X. Chen F. (2025). The role of Enoyl reductase in the monacolin K biosynthesis pathway in *Monascus spp*. J. Fungi 11:199. doi: 10.3390/jof11030199, PMID: 40137237 PMC11943018

[ref47] Ye F. Zhang C. Liu S. Liu X. Liu J. Guo T.. (2024). Optimization of medium compositions and X-ray irradiation to enhance monacolin K production by *Monascus purpureus* in submerged fermentation. Process Biochem. 141, 50–60. doi: 10.1016/j.procbio.2024.03.006

[ref48] Yuan Y. J. Gao D. Q. Ma L. Z. Mao L. J. Mao R. S. Meng J.. (2020). Present status of HIRFL complex in Lanzhou. J. Phys. Conf. Ser. 1401:012003. doi: 10.1088/1742-6596/1401/1/012003

[ref49] Yuan X. Gao S. Tan Y. Cao J. Yang S. Zheng B. (2023). Production of red yeast rice rich in monacolin K by variable temperature solid fermentation of *Monascus purpureus*. RSC Adv. 13, 27303–27308. doi: 10.1039/d3ra04374f, PMID: 37705986 PMC10496031

[ref50] Zhang Y. Chen Z. Wen Q. Xiong Z. Cao X. Zheng Z.. (2020). An overview on the biosynthesis and metabolic regulation of monacolin K/lovastatin. Food Funct. 11, 5738–5748. doi: 10.1039/d0fo00691b, PMID: 32555902

[ref51] Zhang C. Chen M. Yang L. Cheng Y. Qin Y. Zang Y.. (2022). Effects of *mokF* gene deletion and overexpression on the monacolin K metabolism yields of *Monascus purpureus*. Appl. Microbiol. Biotechnol. 106, 3069–3080. doi: 10.1007/s00253-022-11913-2, PMID: 35435455

[ref52] Zhang C. Liang J. Zhang A. Hao S. Zhang H. Zhu Q.. (2019). Overexpression of monacolin K biosynthesis genes in the *Monascus purpureus* Azaphilone polyketide pathway. J. Agric. Food Chem. 67, 2563–2569. doi: 10.1021/acs.jafc.8b05524, PMID: 30734557

[ref53] Zhang C. Sun Q. Yang L. Ablimit A. Dong H. Wang H.. (2024). Mutation breeding of Monascus to produce a high yield of Orange pigment and low Citrinin content using the ARTP method. J. Fungi 10:553. doi: 10.3390/jof10080553, PMID: 39194879 PMC11355741

[ref9002] Zhang B. B. Xing H. B. Jiang B. J. Chen L. Xu G. R. Jiang Y.. (2018). Using millet as substrate for efficient production of monacolin K by solid-state fermentation of Monascus ruber. Journal of Bioscience and Bioengineering 125, 333–338. doi: 10.1016/j.jbiosc.2017.10.011 29157871

[ref54] Zhang R. R. Zhou L. H. Xie L. Y. Lu L. Q. Zhou H. Yang Y.. (2025). Metabolite profiling and adaptation mechanisms of under pH stress. Front. Microbiol. 16:1576132. doi: 10.3389/fmicb.2025.1576132 40236484 PMC11998282

[ref55] Zhu M.-M. Liu E.-Q. Bao Y. Duan S.-L. She J. Liu H.. (2019). Low concentration of corn steep liquor promotes seed germination, plant growth, biomass production and flowering in soybean. Plant Growth Regul. 87, 29–37. doi: 10.1007/s10725-018-0449-6

[ref56] Zhu C. Xu Y. Wang D. (2025). Magnesium ions enhance biogenic amine degradation by Pichia kudriavzevii MZ5: insights from transcriptomics and novel recombinant enzyme expression. Int. J. Biol. Macromol. 306:141617. doi: 10.1016/j.ijbiomac.2025.141617, PMID: 40024410

